# Monitoring the colonization and infection of legume nodules by *Micromonospora* in co-inoculation experiments with rhizobia

**DOI:** 10.1038/s41598-017-11428-1

**Published:** 2017-09-08

**Authors:** Patricia Benito, Pablo Alonso-Vega, Carolina Aguado, Rafael Luján, Yojiro Anzai, Ann M. Hirsch, Martha E. Trujillo

**Affiliations:** 1 Departamento de Microbiología y Genética, Edificio Departamental, Campus Miguel de Unamuno, Universidad de Salamanca, Salamanca, Spain; 2 Synaptic Structure Laboratory, Instituto de Investigación en Discapacidades Neurológicas (IDINE), Departamento Ciencias Médicas, Facultad de Medicina, Universidad Castilla-La Mancha Albacete, Ciudad Real, Spain; 3 Department of Microbiology, Faculty of Pharmaceutical Sciences, Toho University, Funabashi, Chiba, Japan; 4 Department of Molecular, Cell and Developmental Biology and Molecular Biology Institute, University of California-Los Angeles, Los Angeles, CA USA

## Abstract

The discovery that the actinobacterium *Micromonospora* inhabits nitrogen-fixing nodules raised questions as to its potential ecological role. The capacity of two *Micromonospora* strains to infect legumes other than their original host, *Lupinus angustifolius*, was investigated using *Medicago and Trifolium* as test plants. Compatible rhizobial strains were used for coinoculation of the plants because *Micromonospora* itself does not induce nodulation. Over 50% of nodules from each legume housed *Micromonospora*, and using 16S rRNA gene sequence identification, we verified that the reisolated strains corresponded to the microorganisms inoculated. Entry of the bacteria and colonization of the plant hosts were monitored using a GFP-tagged Lupac 08 mutant together with rhizobia, and by using immunogold labeling. Strain Lupac 08 was localized in plant tissues, confirming its capacity to enter and colonize all hosts. Based on studying three different plants, our results support a non-specific relationship between *Micromonospora* and legumes. *Micromonospora* Lupac 08, originally isolated from *Lupinus* re-enters root tissue, but only when coinoculated with the corresponding rhizobia. The ability of *Micromonospora* to infect and colonize different legume species and function as a potential plant-growth promoting bacterium is relevant because this microbe enhances the symbiosis without interfering with the host and its nodulating and nitrogen-fixing microbes.

## Introduction

Nitrogen-fixing nodules are unique structures, which are formed on the roots of legume and actinorhizal plants to establish a nitrogen-fixing symbiosis with either rhizobia or *Frankia*. One striking feature of the legume–rhizobial symbiosis is its high level of specificity in that a rhizobial strain nodulates and fixes nitrogen with usually only a limited number of host plant species. This specificity is determined by several stages of chemical signaling between the symbiotic partners^1^.

Nodular tissues and their carbohydrate supplies are an excellent habitat for bacteria, not only for nitrogen-fixing rhizobia, but also for many other microbes^2^. Numerous reports about the presence of non-rhizobial microorganisms associated with nitrogen-fixing nodules have been published. Muresu and co-workers^3^ described the isolation of at least 12 different taxa from surface-sterilized nodules of several wild legumes. The bacterial genera identified included *Bacillus*, *Pseudomonas*, *Rhizobium*, *Xanthomonas*, and members of the family *Enterobacteriaceae*. Many members of the phylum *Actinobacteria* have also been isolated from legume nodule tissues including *Agromyces* and *Microbacterium* spp^3–5^., *Curtobacterium* ^6^, and *Micromonospora* ^7–12^.

*Micromonospora* is a Gram-positive bacterium characterized by filamentous growth and spore production on substrate mycelium. It is aerobic and many strains produce carotenoid pigments. *Micromonosporae* have been mainly reported from soil and aquatic environments where they are thought to be involved in organic matter turnover, especially of cellulose^13, 14^. In 2007, we reported the first isolation of *Micromonospora* strains from nitrogen-fixing nodules of the wild legume *Lupinus angustifolius* ^8^. Since then, our research groups have focused on the ecology of *Micromonospora* and its interactions with plants, and we have documented the distribution of this bacterium in a wide range of legumes and actinorhizal plants^10, 12, 15^. Other *Micromonospora* strains isolated from other sources such as calcareous soil, have also been reported to promote the growth of *Phaseolus vulgaris* by solubilizing phosphate^16^.

Most studies of beneficial plant-microbe interactions focus on a single plant-microbe partnership at a time. However, simultaneous infection with rhizobia and a number of other bacteria also present in nodules enhances nodulation and plant growth in a wide variety of legumes. Current data suggest that although *Micromonospora* species do not induce nodules or fix nitrogen in association with a host plant, they provide many benefits to the plant by increasing the number of nodules, enhancing aerial growth and nutrient uptake^17–19^. Studying ways to augment plant productivity through the use of beneficial microbes will increase our knowledge of plant-microbe interactions, which has deep implications for agriculture and biotechnology.

Plant growth is promoted by various mechanisms, including improved access to and uptake of minerals and nutrients, amelioration of soil toxicity, release of growth-stimulating phytohormones as well as modulation of plant hormone production, acquisition of nitrogen and phosphate via symbioses, and/or enhancement of the effects of symbioses^20^. Studies based on *Micromonospora* strains isolated from alfalfa nodules suggest that the actinobacteria contribute to the nutritional efficiency of this legume^17^, and our experimental data showed that *Micromonospora lupini* Lupac 08 is a plant growth-promoting bacterium (PGPB). The localization of several genes in the genome that are involved in plant growth promotion, such as for the production of siderophores, phytohormones, the degradation of chitin (for biocontrol), and the biosynthesis of trehalose all contribute to the welfare of the host plant^18^.

Until now, most inoculation experiments to analyze the effect of *Micromonospora* on a host plant and its interaction with rhizobia have been carried out using the same plant species from which the strains originated. However, no information is available as to whether any specificity exists in the *Micromonospora*-legume interaction. Thus, the present study was designed to test the capacity of *M*. *lupini* Lupac 08 and *M*. *saelicesensis* Lupac 09^T^ (both isolated from *Lupinus angustifolius*) to enter *Medicago* and *Trifolium* nodules and to obtain information about the location of *Micromonospora* with nodule tissues. Host-associated rhizobial strains were used for coinoculation of *Micromonospora* onto legume plants to induce nodulation and facilitate the entry of this microbe into plant tissues. *Micromonospora* strains, which were identified by 16 S rRNA gene sequencing, were re-isolated from the resulting nodules. Finally, *Micromonospora* localization within the plant tissues was performed using green fluorescent protein and immunogold labeling to localize strain Lupac 08 within the plant cells. The results indicate that *Micromonospora* has the capacity to enter and colonize additional legumes beyond the legume host from which it was isolated originally, and strongly suggest that this non-specific, but beneficial PGPB can be used to enhance productivity of a wide range of nodulating plants.

## Results

In a previous study, we showed that *Micromonospora* strains isolated from nitrogen-fixing nodules contribute to plant development and health by acting as PGPB^18^. In the present work, our aim was to determine if *Micromonospora* when grown *in vitro* could re-colonize the plant from which it was isolated and also whether this process is host-specific. In addition, we searched for information about the entry and localization of *Micromonospora* within the nodules. To this end, three different plants, *L*. *albus*, *M*. *sativa*, and *T*. *repens*, were grown axenically and co-inoculated not only with the corresponding symbiont to induce nodulation, but also with *Micromonospora lupini* Lupac 08 or *M*. *saelicesensis* Lupac 09^T^, which were originally isolated from *L*. *angustifolius* nodules. Alfalfa and clover nodules are of the indeterminate type, as are lupine nodules. However, the latter develops several lateral meristems, which continue to undergo cell divisions and eventually form lobes that wrap around the parent root, giving the nodules a “collared” appearance^21^.

### Recovery of *M*. *lupini* Lupac 08 and *M*. *saelicesensis* Lupac 09^T^ from *Lupinus*, *Medicago*, and *Trifolium* nodules

The number of *Micromonospora* colonies recovered from a single nodule of the inoculated plants varied, ranging from zero to more than 300. However, on average, most of the nodules yielded between 1–4 CFUs, and about 60% of the 60 nodules screened contained *Micromonospora*. Regarding the success of infection, the most colonies were isolated from lupine nodules, with a slightly lower number of CFUs from the clover and alfalfa nodules. PCR with 16 S rRNA primers confirmed that the isolates were *M*. *lupini* Lupac 08 and *M*. *saelicesensis* Lupac 09^T^. Overall, strain Lupac 09^T^ was more effective, with 77% of the nodules screened harboring this *Micromonospora* strain.

### Localization of *Micromonospora* in lupine

**Figure 1:**
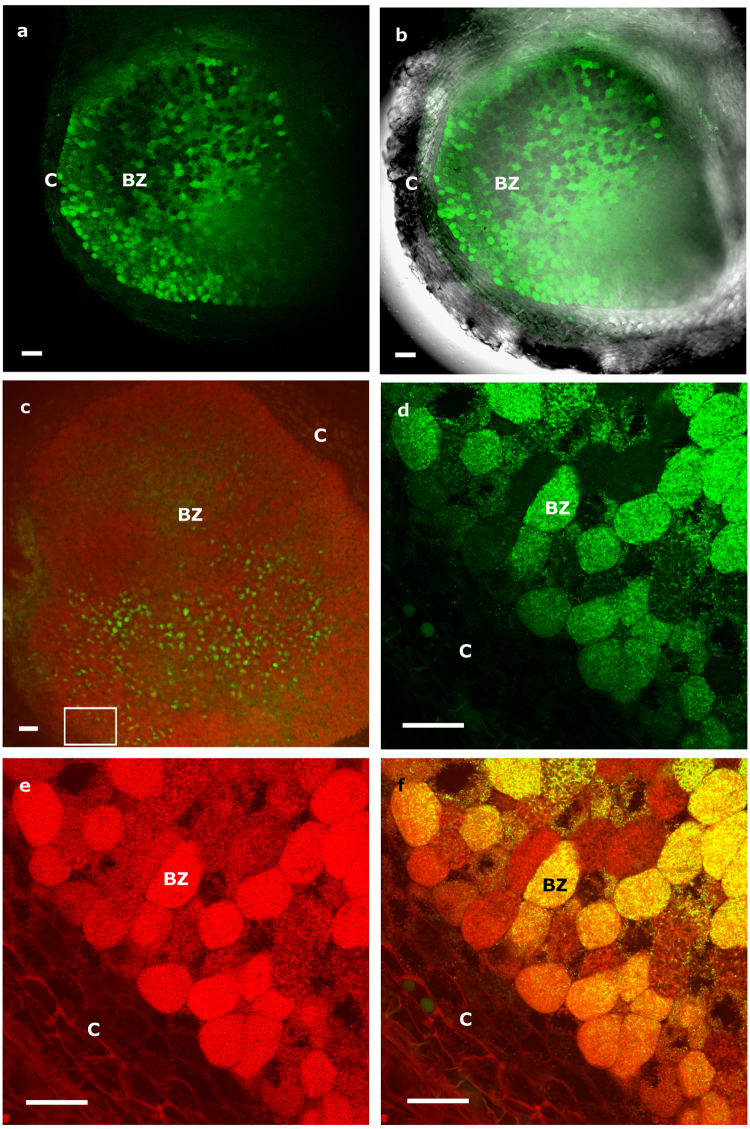
Longitudinal nodule sections of *Lupinus albus* coinoculated with *Bradyrhizobium* sp. CAR08 and *Micromonospora* ML01-*gfp* (21 dpi). (**a**) Green fluorescence signal captured by CLSM of infected cells containing *Micromonospora* ML01-*gfp*. **(b**) Overlay of light and fluorescence images of the nodule section. (**c**) Green fluorescence localization of ML01-*gfp* in a nodule section stained with propidium iodide and viewed by CLSM. (**d**) Higher magnification image captured with the green channel. (**e**) Higher magnification image captured with the red channel. (**f**) Composite image of both channels. The white rectangle in image c shows the area where images d-f were captured. C, cortex; BZ, bacteroid zone; dpi, days post inoculation. Bars: 100 µm (**a**, **b**, **c**); 40 µm (**d**, **e**, **f**).

The nodules produced by all coinoculated *Lupinus* plants were pink in color, indicating effective nitrogen fixation. Several root nodules (~35–40 dpi) were randomly selected and longitudinally sectioned to localize *M*. *lupini* ML01-*gfp* cells by CLSM. The bacteria were successfully localized in several zones of the nodule, but they were especially prevalent in the infection and bacteroid zones (BZ; terminology of ref. 22) (Fig. 1a,b). Although González-Sama *et al*.^23^ reported that uninfected cells were not present in the central BZ of *L*. *albus* nodules, *Micromonospora* cells were observed in those host cells that appeared devoid of *Bradyrhizobium*. This was more obvious in longitudinal nodule sections that were counterstained with propidium iodide to differentiate *Bradyrhizobium* sp. CAR08 from the *gfp*-tagged *Micromonospora* (Fig. 1c–f). As expected, the bradyrhizobia occupied the majority of the cells within the nodule tissue, and were clearly seen in the infection and bacteroid zones of the nodule, whereas *Micromonospora* cells were observed in fewer host cells, which were interspersed among the *Bradyrhizobium*-infected cells (Fig. 1c). The presence of both bacteria in the same cell was detected as yellow fluorescence (Fig. 1f) due to the coincidence of the green and red fluorescence (Fig. 1d,e).

**Figure 2:**
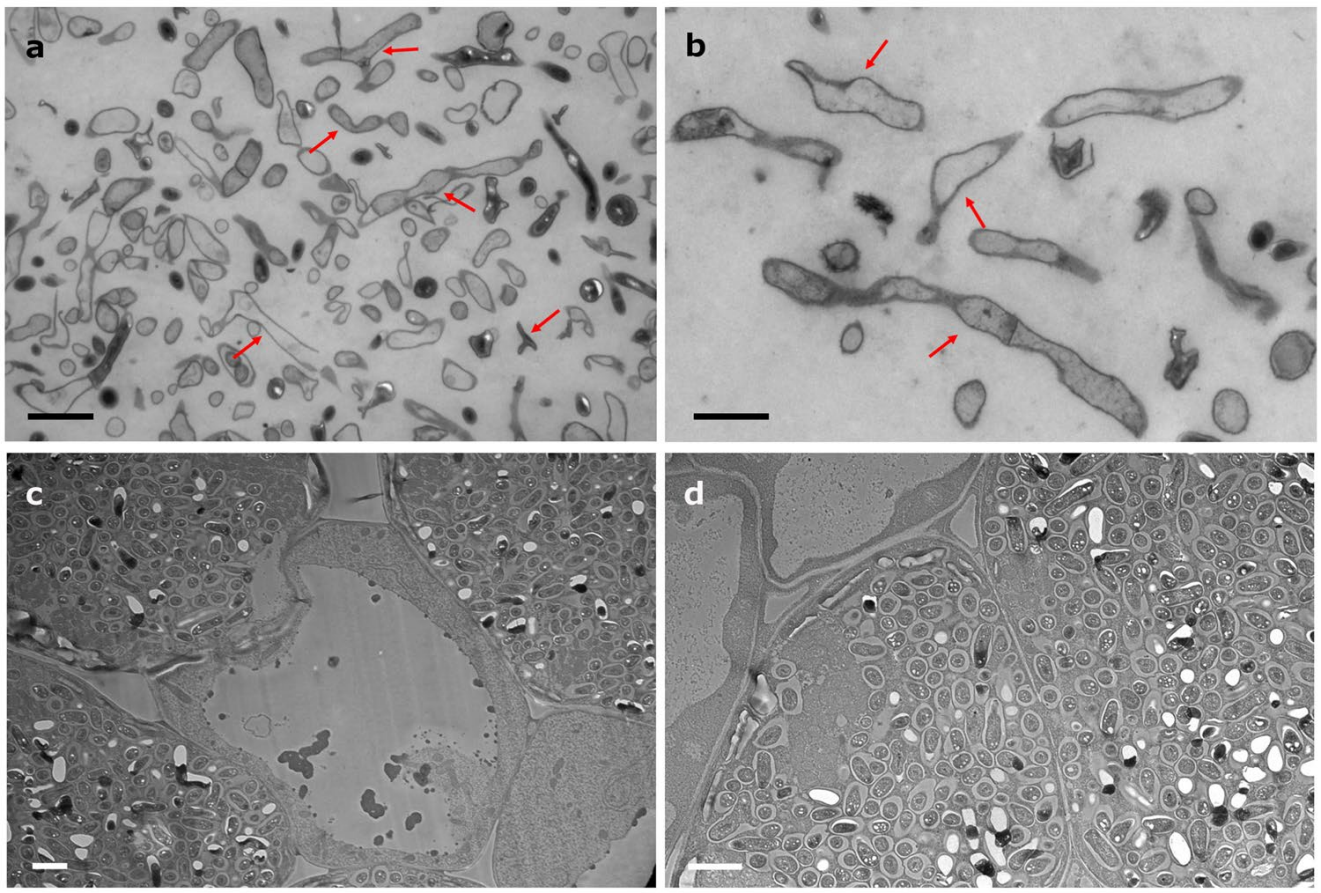
Transmission electron micrographs of *Micromonospora* pure cultures and nodular tissue infected with *Bradyrhizobium*. **(a**,**b)** *Micromonospora* ML01-*gfp* pure cultures (arrows, polymorphic *Micromonospora* cells). **(c**,**d)** Lupine nodule tissue infected with *Bradyrhizobium* sp. CAR08 only. Bar: 2 µm (**a**, **c**, **d**); 1 µm (**b**).

Immunogold microscopy was used to confirm the nodule occupancy of the *Micromonospora* cells. Pure cultures of the bacteria were observed by TEM (Jeol 1010, Japan) for comparison purposes. As expected, *Micromonospora* cells were seen as branched filaments or rod-shaped structures that corresponded to longitudinal and transverse sections, respectively (Fig. 2a,b). TEM preparations of nodules inoculated with *Bradyrhizobium* sp. CAR08 only were sectioned and served as controls. Figures 2c,d illustrate infected cells containing bacteroids within their symbiosomes as well as uninfected plant cells.

**Figure 3:**
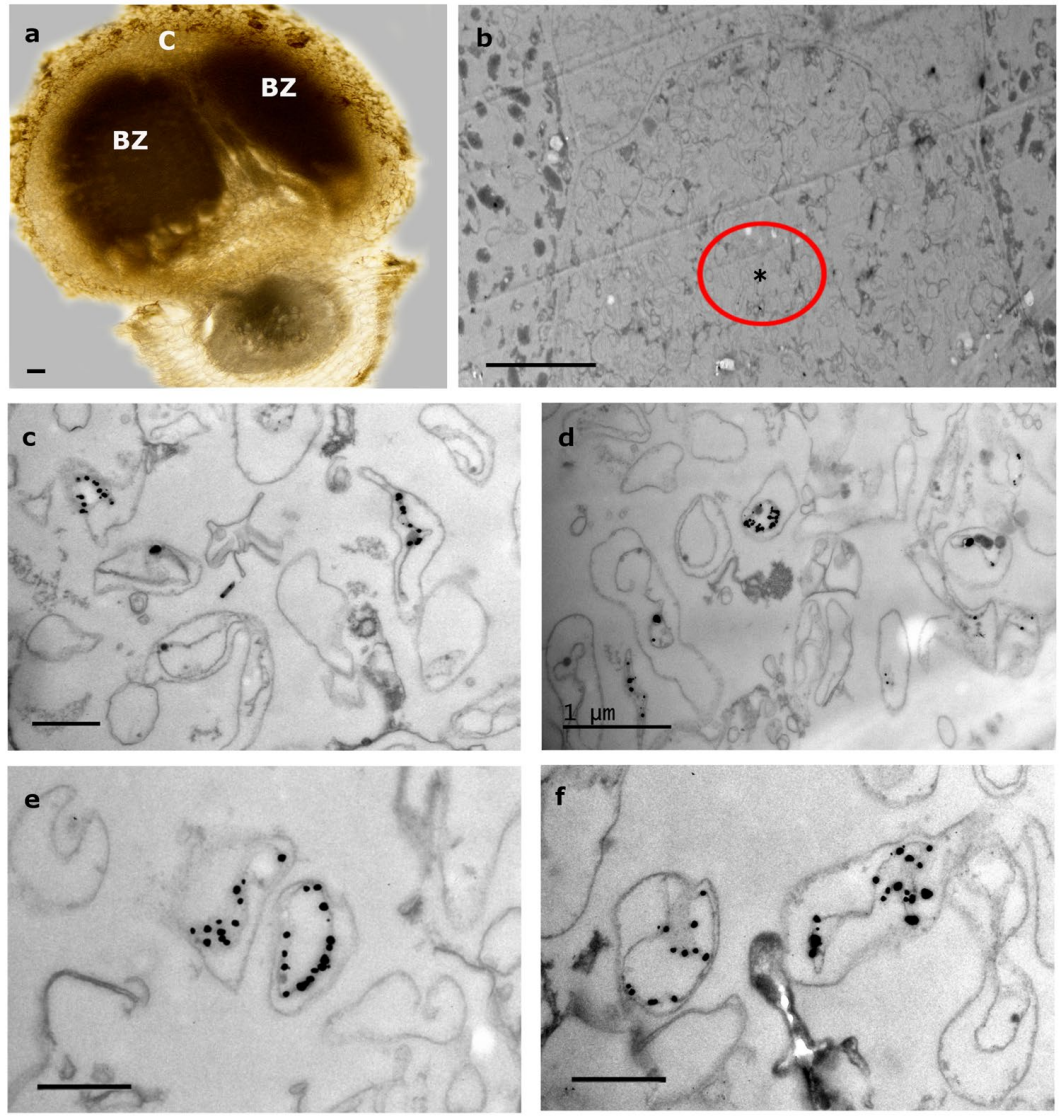
Immunoelectron microscopic images of lupine nodules infected with *Bradyrhizobium* CAR08 and *Micromonospora* ML01*-gfp* (21 dpi). (**a**) Light micrograph of a longitudinal nodule section. (**b)** Detail of an “empty” cell between two infected cells that contain bacteroids. (**c–f**) Labeled *Micromonospora* cells found in the area marked with an encircled asterisk in 3b. Bars: (**a**) 100 µm; (**b**) 10 µm; (**d**) 1 µm; 500 nm (**c, e, f**). C, cortex; BZ, bacteroid zone; dpi, days post inoculation.

Nodule sections obtained from plants that were coinoculated exhibited a structure similar to the control nodules with zones corresponding to the nodule cortex (C) and the infection and bacteroid zones (BZ) (Fig. 3a). Within these zones, bacteria-containing plant cells were seen in addition to what appeared to be uninfected plant cells (Fig. 3b). *Micromonospora* hyphae were usually found in the latter cells, which at low magnification gave the impression of being “empty”. Figure 3b shows a *Micromonospora*-containing nodule cell, which is flanked by two host cells containing *Bradyrhizobium* bacteroids; the areas where the *gfp*-ML01 strain was found are marked with an asterisk enclosed by a circle. Within these cells, immunogold-labeled structures that resembled the cells of *Micromonospora* were observed (Fig. 3c–f). These structures were similar to those found in the pure culture preparations (Fig. 2a,b), but were not detected in the nodules inoculated with *Bradyrhizobium* only. Unlike many rhizobia that undergo physical changes such as a marked increase in cell size and morphological transformation from rod-shaped to branched cells^24^, the *Micromonospora* cells did not show any drastic morphological changes and many labeled cells resembled those observed in pure culture. In some cases, both types of bacteria were seen within the same plant cell (Fig. 1), but *Micromonospora* was always found in lower numbers compared to the *Bradyrhizobium* bacteroids.

### Effect of *Micromonospora* on the root hairs of *Medicago* and *Trifolium*

**Figure 4:**
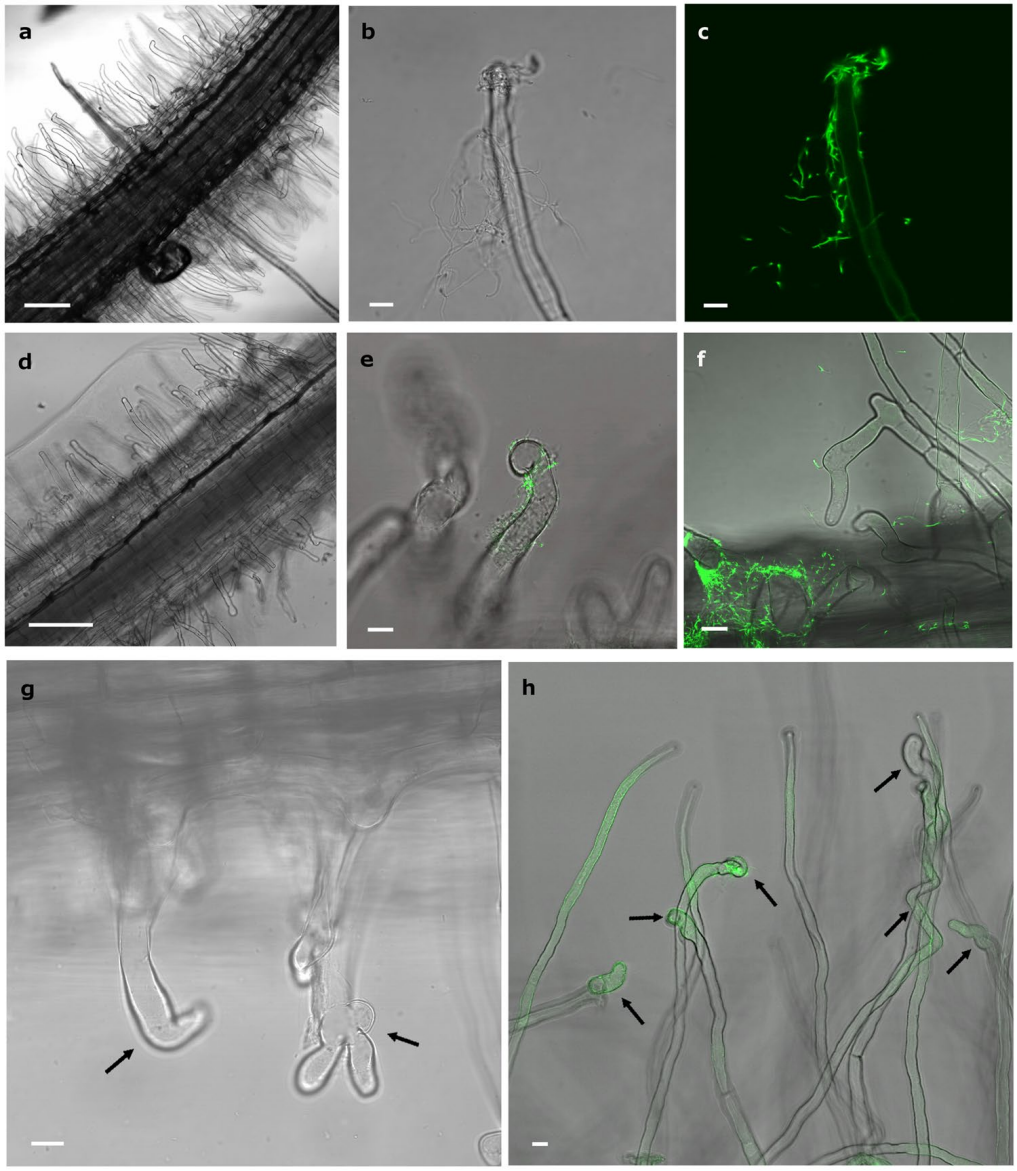
Effect *of Micromonospora* on the root hairs of *Trifolium* and *Medicago*. **(a,d)** Control uninoculated plants of *Trifolium* and *Medicago* respectively. **(b**,**c**) Light and CLMS micrographs of *Micromonospora* cells attached to a *Trifolium* hair root 3 dpi. (**e**,**f**) CLMS images of *Micromonospora* attached to *Medicago* root hairs. **(g**) *Medicago* and (**h**) *Trifolium* root hairs showing different deformations (arrows) 5 dpi with *Micromonospora*. Bars: (**a**) 100 µm; (**b, c, h**) 8 µm; (**d**) 200 µm; (**e**, **f**, **g**) 10 µm; dpi, days post infection.

The ability of *Micromonospora lupini* Lupac 08 to infect legumes other than *L*. *albus* was investigated. *Medicago and Trifolium* plants inoculated with *Micromonospora gfp*-labeled ML01 were observed under light and confocal microscopy. Root hair deformations were observed as early as 2–3 days after inoculation. In both plants, *Micromonospora* was observed attached to the root surfaces (Fig. 4b,c,e,f). Root hairs deformed branching into Y and L shapes (Fig. 4g), developing zig-zag forms and/or swollen root hair tips (Fig. 4h). Root hairs of uninoculated *Trifolium* (Fig. 4a) and *Medicago* (Fig. 4d) control plants exhibited no deformation.

### Infection of *Medicago* and *Trifolium* root nodules by *Micromonospora*

**Figure 5:**
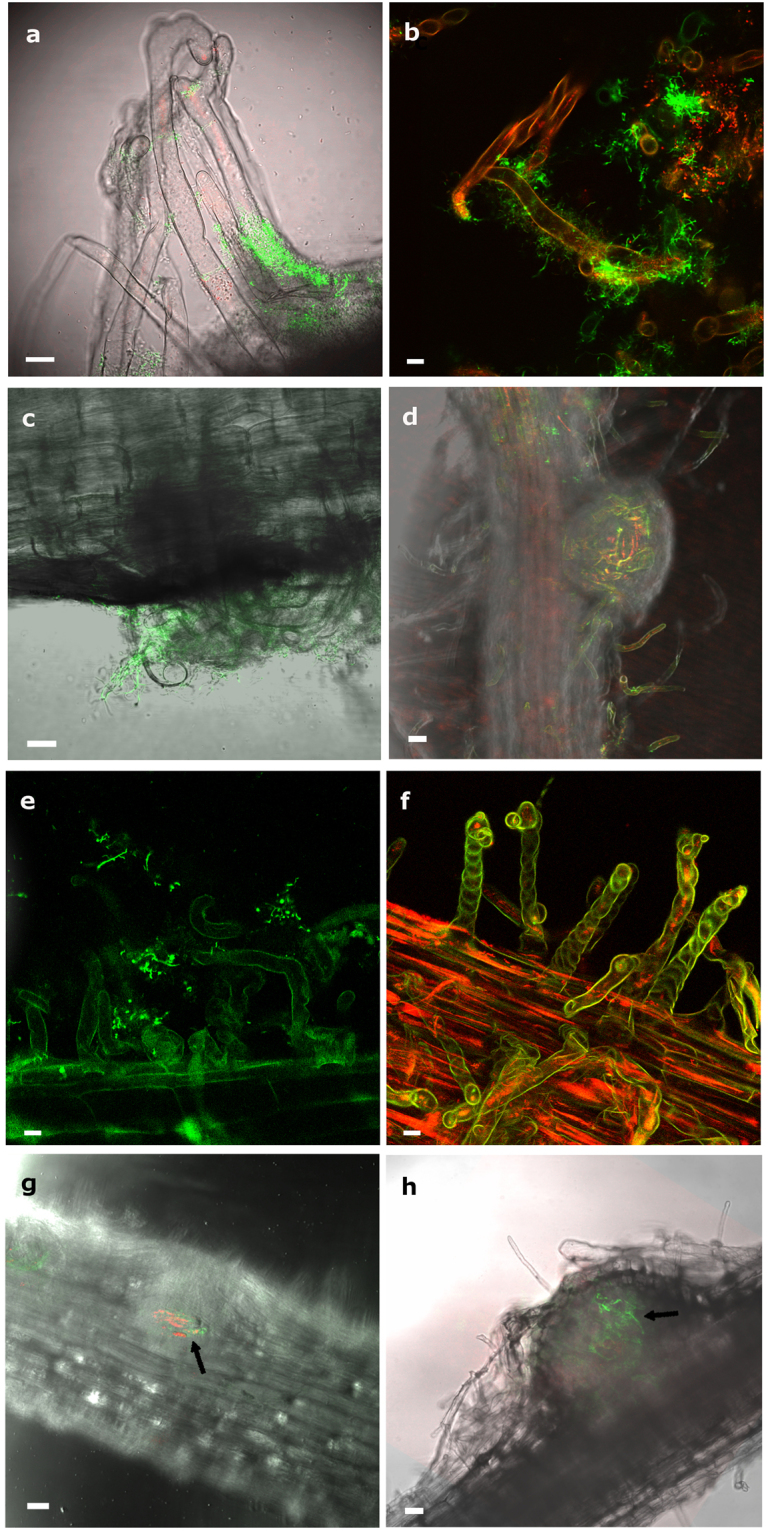
Infection and colonization of *Trifolium* and *Medicago* by *Micromonospora* ML01-*gfp* co-inoculated with strains *Rhizobium* sp. E11-*mCh* and *Sinorhizobium* sp. Rm1021-*mCh* respectively, and observed by CLSM. (**a**) *Trifolium* root tip deformations observed 3 dpi and surrounded by *Micromonospora* ML01-*gfp*. (**b**) *Micromonospora* and *Rhizobium* sp. co-localized on the root hairs. (**c**) Nodule primordium and deformed root hairs observed in *Trifolium* 5 dpi. (**d**) Young *Trifolium* nodule observed 11 dpi. The fluorescent bacteria are visible within the internal tissues of the nodule. **(e)** Attachment of *Micromonospora* to *Medicago* root tips showing deformations 6 dpi. (**f**) *Medicago* root hair tips forming spiral shapes 12 dpi. (**g**,**h)** *Medicago* nodules at 11 and 13 dpi with green and red fluorescence signals showing strings of bacteria. Because of the thickness of the tissue, the nodules themselves are slightly out of focus. For details see text. Bars: 8 µm (**a**, **b**); 10 µm (**c, e, f**); 75 µm (**g**, **h**). dpi, days post infection.

To determine the capacity of *Micromonospora* to penetrate and infect nodular internal tissues, *Medicago* and *Trifolium* plants were co-inoculated with the appropriate *mCherry*-tagged nitrogen-fixing rhizobia (*Sinorhizobium* Rm1021-*mCh* and *Rhizobium* sp. E11-*mCh*, respectively) as well as *Micromonospora* ML01-*gfp*. The plants were monitored by light and CLSM microscopy, every two days until approximately 25 dpi. Root tip deformations appeared 2 days after bacterial inoculation in *Trifolium* (Fig. 5a,b) whereas the changes in root hair deformation did not occur in *Medicago* until 6 dpi (Fig. 5e,f). In both sets of plants, *Micromonospora* surrounded the youngest regions of the root. Although green autofluorescence was detected within *Medicago* tissues once the nodules formed, *Micromonospora* cells were also clearly attached to the root hairs (Fig. 5e). Differences in root hair morphology were observed between the two plant species; most *Trifolium* root hairs were branched or club-shaped 2–4 dpi (Fig. 5a,b) whereas *Medicago* root hairs became spiral in shape, but not until 12 dpi (Fig. 5f).

Nodule primordia were visible 3–5 dpi in *Trifolium* and after 7–9 days in *Medicago*. In both plants, the nodule primordia were covered with deformed root hairs and *Micromonospora* cells were attached to the hairs (Fig. 5c). *Micromonospora* ML01-*gfp* and *Rhizobium* E11-*mCh* were readily visible inside *Trifolium* young nodules 11 dpi, and both bacteria were co-localized as indicated by the yellow fluorescence, supporting the conclusion that both microorganisms were present (Fig. 5d). Comparably aged *Medicago* nodules were thicker, however, and the green and red fluorescence corresponding to *Micromonospora* ML01-*gfp* and *Sinorhizobium* SM1021-*mCh*, respectively, was detected in the intact nodules (Fig. 5g,h).

Nodules were well developed in both plants after 15–20 days and fresh samples had a pink color indicating that nitrogen fixation was taking place. Mature nodules (~20 dpi) of both plant species exhibited the typical indeterminate structure: meristematic, infection, bacteroid, and senescent zones were identified.

**Figure 6:**
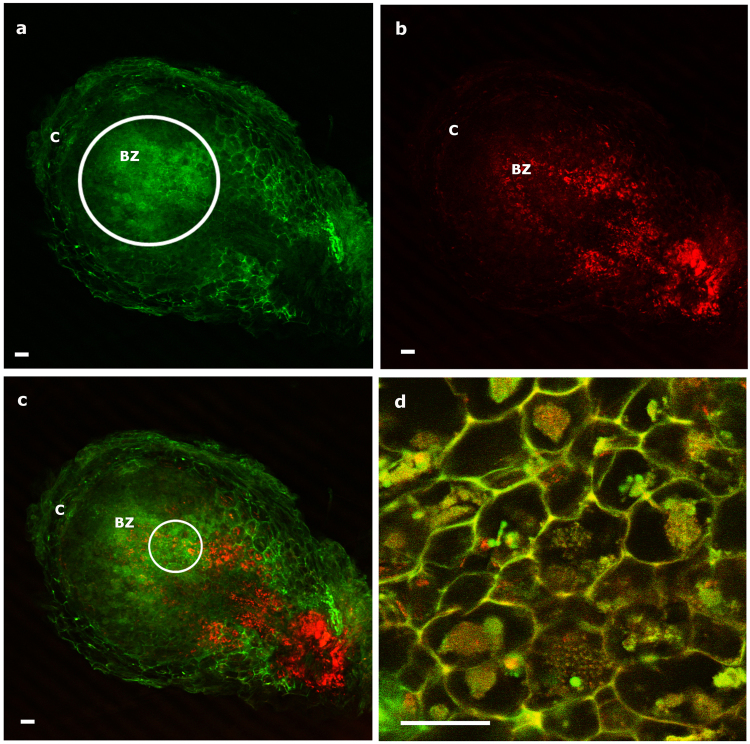
*Trifolium* longitudinal nodule section (20 dpi) showing the distribution of infected cells after co-inoculation with *Micromonospora* ML01-*gfp* and *Rhizobium* E11-*mCh* captured by CLSM. (**a**) Image obtained with the green channel for the localization of *Micromonospora*. The circled area indicates fluorescence emitted by *Micromonospora* concentrated in the bacteroid zone. (**b**) Image captured with the red channel for the localization of *Rhizobium*. (**c**) Combination of images a and b. (**d**) Detail of infected zone showing the co-localization of *Micromonospora* and *Rhizobium* in the host cells. The white circle in 6c shows the area where image (**d**) was captured. Bars: 60 µm (**a**–**c**); 20 µm (**d**). dpi, days post inoculation; C, cortex; BZ, bacteroid zone.

Longitudinal sections of 20-day old *Medicago* and *Trifolium* nodules were obtained for localizing *Micromonospora* by CLSM. In *Trifolium*, a green fluorescence signal (excitation 488-nm and 515- to 560-nm emission) was observed in the infection zone just below the meristematic area. Fluorescence expressed by *Micromonospora* ML01-*gfp* was clearly observed within the plant cells whereas the uninfected cells showed no fluorescence apart from the autofluoresence emitted by the plant (Fig. 6a). *Rhizobium* sp. E11-*mCh* cells exhibited a bright red fluorescent signal (620 nm excitation and 620–660 nm emission) in the infected, bacteroid, and senescent zones of the nodule (Fig. 6b). Co-localization of both bacteria is shown in Fig. 6c. For the most cases, the microbes were found in the infection zone, whereas in other instances, *Rhizobium* E11-*mCh* was the only occupant, especially in the senescent zone. Both bacteria were also located within the same host cell as indicated by yellow fluorescence (Fig. 6d).

**Figure 7:**
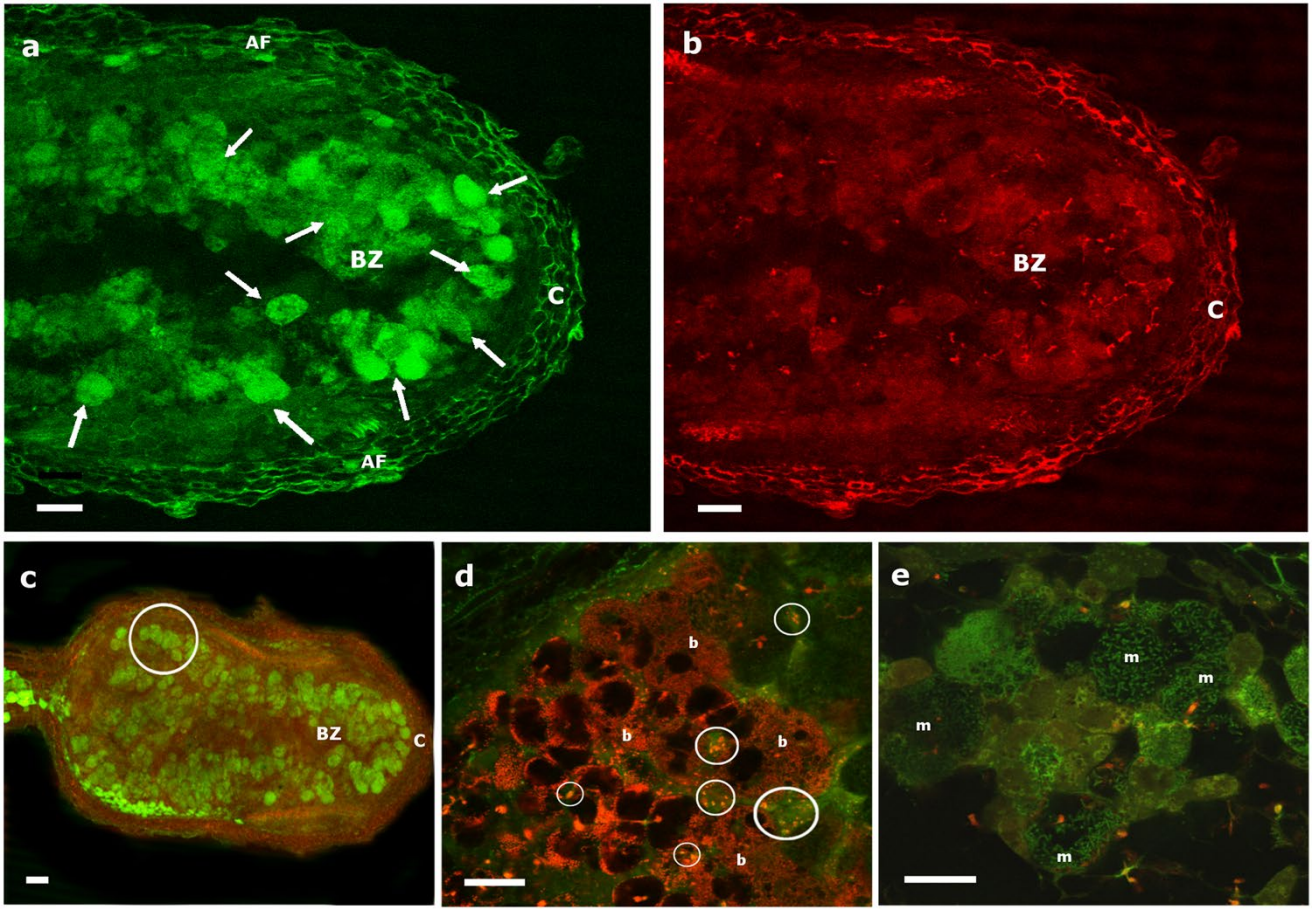
*L*ongitudinal sections of a *Medicago* 20 dpi nodule showing the distribution of cells infected with *Micromonospora* ML01-*gfp* and *Sinorhizobium* Rm1021-*mCh* captured by CLSM. (**a**) Detail of a nodule tip captured with the green channel for the localization of *Micromonospora*. Arrows indicate fluorescence emitted by *Micromonospora* to differentiate from autofluorescence emitted by the plant. (**b**) Detail of a nodule tip captured with the red channel for the localization of *Mesorhizobium* Rm1021-*mCh*. (**c**) Composite image showing the distribution of green and red fluorescence. (**d**) Bacteroids occupying several plant cells, small circles show areas occupied by both bacteria. (**e**) *Micromonospora* hyphae inside host plant cells. The white circle in image 7c shows the area where images d and e were captured. Bars: 75 µm (**a**); 30 µm (**b**–**e**). dpi, days post inoculation; C, cortex; BZ, infection zone; AF, autofluorescence; b, bacteroids; m, *Micromonospora*.

For *Medicago*, *Micromonospora* was localized across the nodule and infected cells were visible in all but the meristematic zone (Fig. 7a). As in *Trifolium* nodules, in some cases, both bacteria occupied the same host cells (Figs. 7c,d). Figure 7a,b show the distribution of the bacteria captured by the corresponding fluorescence channels (green for *Micromonospora* and red for *Sinorhizobium*). A close up of the nodular tissue permitted a clear visualization of *Micromonospora* ML01-*gfp* cells (Fig. 7e).

## Discussion

Legumes are widely recognized for their agricultural importance and their capacity to form nitrogen-fixing nodules in symbiosis with bacteria, generically known as rhizobia. However, nodular tissues are an excellent habitat for microorganisms because they are richer in nutrients compared to other plant organs such as roots. Indeed the number of studies reporting the isolation of non-rhizobial bacteria from this niche is increasing^2, 8, 10, 12, 25^.

To our knowledge, this is the first report for a legume that describes in detail the surface interaction and colonization process of a non-rhizobial bacterium together with the nitrogen-fixing rhizobia. Our results demonstrate that *Micromonspora* is localized within nodules of three different legumes and strongly suggests that a non-specific relationship takes place between *Micromonospora* and the plant. We base this conclusion on data showing that a *Micromonospora* strain isolated from lupine colonizes *Medicago* and *Trifolium* as well as *Lupinus*, suggesting that this actinobacterial strain has a broad host range. Also, *Micromonospora* has been isolated from a diversity of legume nodules, including *Coriaria myrtifolia* ^7^; *Pisum sativum* ^9, 11^, and *Medicago sativa* ^17^. The ability of *Micromonospora* to infect different legume species contrasts with the symbiotic interactions between rhizobia and legumes and *Frankia* and actinorhizal plants, both of which are more restrictive^26, 27^. However, not all nodules housed both strains. The reason for this is not known, but the results are similar to our findings with wild, collected nodules. Further studies including additional legume species are necessary to determine the full capacity of *Micromonospora* to infect plants.

The plants did not show any negative effects related to the presence of *Micromonospora* and the nitrogen fixation process did not appear to be altered. The results also indicated that the different rhizobia were not inhibited by coinoculation with the actinobacterium and that bacteroid development proceeded normally. Indeed, the growth of the coinoculated plants was better, such that in some cases a larger number of nodules per plant resulted^18^. Interestingly, when *Bradyrhizobium* and *Micromonospora* were grown as a co-culture, no growth inhibition was observed by either bacterium even though strain Lupac 08 contains several genes that code for bacteriocins^18^. These genes may not be expressed when the two bacteria interact under the conditions tested, but more studies are needed.

The systematic isolation of *Micromonospora* cells from nitrogen-fixing legume and actinorhizal nodules and the application of fluorescent *in situ* hybridization (FISH) and TEM techniques, presented strong evidence that *Micromonospora lupini* Lupac 08 was a normal inhabitant of internal root nodule tissues and suggested a close interaction between the host plant and the bacterium^10–12, 28, 29^. In the present work, unambiguous microscopic localization of *Micromonospora lupini* Lupac 08 was accomplished using a combination of tagged reporter genes and immunogold labeling. Furthermore, we have demonstrated that *Micromonospora* not only re-enters its original host, *Lupinus* sp., but also interacts with other legumes such as *Medicago* and *Trifolium*. In all plant samples studied, a plant growth-promoting effect as previously reported was confirmed^18, 28^ and these results are in line with those reported by other researchers^19, 30, 31^.

By monitoring the colonization process, information about the distribution of *Micromonospora* in *L*. *albus*, *Trifolium*, and *Medicago* nodules was obtained. In all cases, the infection zone was the main area where *Micromonospora* was found and the place where both bacteria were observed occupying the same plant cell. These results strongly suggest a tripartite interaction and the coexistence of non-rhizobial bacteria within nodule tissues^3, 30^ although at present a specific function cannot be attributed to *Micromonospora*. Genomic analysis of strain Lupac 08 has revealed several features related to plant growth promotion including the production of siderophores, phytohormones and other secondary metabolites, all of which may be involved in growth enhancement^18^.

Furthermore, as compared to control plants inoculated with *Rhizobium* or *Mesorhizobium* only, on average, the nodules appeared and developed 1–2 days earlier on the coinoculated plants. It was previously reported that legumes coinoculated with their compatible nitrogen fixer and associated “helper” *Micromonospora* developed a greater number of nodules in *Lupinus, Medicago*, and *Trifolium* ^18, 19^. Similar results were reported for actinorhizal plants and other “helper” bacteria^31–33^. In the latter cases, however, these bacterial growth promoters were not considered endophytes because they were isolated from the external plant tissues or from the rhizosphere. In the present study, the *Micromonospora* strains tested were isolated from the internal root nodule tissues^8^.

Another question that remains to be answered is whether *Micromonospora* can enter alone or whether the presence of rhizobia is necessary for the bacterium to gain access to the internal tissues. An interaction between the bacterium and the plant’s root hairs was observed, but further studies are necessary to identify the molecules involved in this communication and whether *Micromonospora* enters the root hair together with the rhizobial strain or via a “crack entry” mechanism. Plant-polymer degrading enzymes are known to play an important role in infection and colonization^34–36^. In this respect, genomic analysis of *M*. *lupini* Lupac 08 revealed an important array of plant cell wall-degrading enzymes, which have been tested for functionality and may be involved in the infection process. Future investigations will be focused on answering some of these questions with the aim of increasing our knowledge of managing microbial communities that interact with plants. Understanding the ecology of these endophytic bacteria and their molecular interactions will have an impact in plant growth and crop yields and therefore in economics and the environment.

## Materials

### Strains, plant media, and culture conditions

The strains and plants used in the present study are listed in Table 1. *Micromonospora* strains were routinely sub-cultured on SA1 agar^37^. Rhizobial strains were maintained on yeast-mannitol agar^38^. *Escherichia coli* strains were grown in Tryptic Soy Broth (TSB) or Luria-Bertani agar (LB). The media were supplemented with the following antibiotics: apramycin (25 µg/ml), kanamycin (60 µg/ml), tetracycline (10 µg/ml), and streptomycin (100 µg/ml), when needed.

**Table 1:** Strains used in the present study.

Strain	Characteristics	Inoculation host
*M*. *lupini* Lupac 08	Wild type	*Lupinus*, *Medicago*, *Trifolium*
*M*. *saelicesensis* Lupac 09^T^	Wild type	*Lupinus*, *Medicago*, *Trifolium*
*S*. *meliloti* Rm 1021	Wild type	*Medicago*
*Rhizobium* sp. E11	Wild type	*Trifolium*
*Bradyrhizobium* sp. CAR08	Wild type	*Lupinus*
*M*. *lupini* ML01-*gfp*	*M*. *lupini* Lupac 08 egfp reporter gene	*Medicago*, *Trifolium*, *Lupinus*
*Sinorhizobium* Rm1021-*mCh*	*S*. *meliloti* Rm 1021(pM7604) carrying an mCherry reporter gene	*Medicago*
*Rhizobium* sp. E11-*mCh*	*Rhizobium* sp. E11 (pM7607) carrying an mCherry reporter gene	*Trifolium*

### Construction of reporter plasmid pSET152-eGFP

The strain *Micromonospora lupini* Lupac 08 was selected as the recipient for introducing a green fluorescent protein (*gfp*) gene for localizing the actinobacteria within the plant. The site-specific integration vector pSET152 containing aac(3)IV(Aprar), ϕC31 *int*, *attP*, and *oriT* was selected as the transconjugation vector for *M*. *lupini* Lupac 08 because it has been shown to integrate into the genome of various actinobacteria including *Micromonospora* ^39^. The presence of the insertion site in the genome of *M*. *lupini* Lupac 08 was confirmed by PCR and sequencing using the primers attBF 5′-GTCGACCCCGGGCCCTGGT-3′ and attBR 5′-GTCGACCACCTACCGGCCGG-3′ under the following PCR conditions: 7 min at 95 °C, 30 cycles of 1 min at 94 °C, 1 min at 65 °C and 3 min at 72 °C, followed by 10 min final extension at 72 °C. Sequencing was carried out on an ABI377 sequencer (Applied Biosystems) using a BigDye terminator v3.0 cycle sequencing kit as supplied by the manufacturer and using the primers attBF and attBR.

The construct eGFP-apramycin promoter *aac(3)IV* was cloned into the plasmid at the EcoRI and NotI restriction sites. The final insert pSET152-eGFP-aac(3)IV(Aprar) was inserted by electroporation into *Escherichia coli* S17.1, the donor strain.

### Conjugation procedure

Intergeneric conjugation between *E*. *coli* S17.1 and *M*. *lupini* Lupac 08 was performed as described previously^39, 40^. Briefly, an overnight culture of *E*. *coli* S17.1 grown in TSB was diluted into fresh medium and incubated for 3–5 h. The cells were harvested, washed twice, and concentrated 10-fold in TSB. Strain Lupac 08 was grown in TSB for 5 days, harvested by centrifugation, washed, and re-suspended in TSB (2:1 v/v). Recipient cells were mixed with *E*. *coli* donor cells (2:1 v/v) and 150 µl were plated on MR0.1 S medium^41^. The plates were incubated at 28 °C for 20 h and then the medium was covered with 1 ml water containing 500 µl of nalidixic acid to inhibit further growth of *E*. *coli* and 1 mg apramycin to select *M*. *lupini* exconjugants. Incubation at 28 °C continued for 2–3 weeks to allow outgrowth of the exconjugants.

### Transformation of rhizobial strains with mCherry constructs

The strains *Sinorhizobium meliloti* Rm1021 and *Rhizobium* sp. E11 were transformed with the plasmids pMP7604 and pMP7607^42^ by conjugation according to standard methods^43^. Conjugation of plasmids was accomplished by mixing the donor *E*. *coli* DH5α containing pMP7604, the helper *E*. *coli* DH5α strain containing pRK2013, and the recipient strains, either *S*. *meliloti* Rm1021 or *Rhizobium* sp. E11.

### Seed germination and infection assays

*Lupinus albus*, *Medicago sativa*, and *Trifolium repens* seeds were germinated axenically. *L*. *albus* seeds were surface-sterilized in 2% (v/v) sodium hypochlorite for 12 min followed by rinsing with sterile distilled water, then placed in Petri dishes containing sterilized moist filter papers, and incubated in the dark. After germination (~3–5 days), seedlings were transferred to pots containing sterile vermiculite and watered with nitrogen-free nutrient solution^44^ as needed. For the *Medicago* and *Trifolium* seeds, these were sequentially immersed in 70% (v/v) ethanol and 2.5% HgCl_2_ (w/v) for 30 s and 2 min respectively, followed by several rinses with sterile distilled water, and then placed on tap-water agar plates in the dark. After germination, seedlings were placed on square Petri dishes (120 × 120 mm) containing nitrogen-free Rigaud and Puppo nutrient agar.

All seedlings were kept in a plant growth chamber with mixed incandescent and fluorescent lighting programmed for a 16 h photoperiod, day-night cycle, with a constant temperature of 21–22 °C, and 50–60% relative humidity. Upon the appearance of the first leaves, the plants were inoculated with the appropriate bacterial suspensions (10^8^ cells per ml). Overall, three different treatments were used: a) plants inoculated with GFP-tagged *Micromonospora lupini* (strain ML01); b) co-inoculation of *Micromonospora* ML01 and the appropriate *Bradyrhizobium*, *Rhizobium* or *Sinorhizobium* strain to induce nodulation (see Table 1), and c) uninoculated plants, which served as negative controls.

### Monitoring bacterial colonization by confocal microscopy

Infected plant roots of *Medicago* and *Trifolium* plants were observed with fluorescence (Nikon Eclipse 80i) and confocal scanning laser (CLSM, Leica TCS model) microscopes 2 days after inoculation and monitored every other day until the nodules were fully developed. Localization of *gfp* and *mCherry* fluorescence in the root and nodule tissues was done using standard filter settings (488 nm excitation and 515 to 560 nm emission for *gfp* expression, and 620 nm excitation and 620–660 nm emission for *mCherry*). Autofluorescence was evaluated by comparing the *gfp* image with the red fluorescence channel (543 nm excitation and > 570 nm emission) and also by comparing the image with uninoculated plants. Coinoculated *Lupinus* plants were grown for 4–5 weeks. For observation, nodules were longitudinally sectioned on a cryostat (Thermo HM560), mounted on glass slides, and viewed by CLSM as described above.

### Immunoelectron microscopy

Strain ML01 was localized in *L*. *albus* nodules via a pre-embedding immunogold technique with antibodies raised against GFP, following the procedures of^45^ and^46^. A vibratome (Leica V1000) was employed to obtain 60 µm-thick sections from fixed (4% paraformaldehyde and 0.1% glutaraldehyde in 0.1 M phosphate buffer [PB]), agarose-embedded nodules. Floating sections were blocked with 10% normal goat serum (NGS) in 0.1 M Tris-buffer containing 0.9% NaCl (TBS) for 1 h. The sections were then labeled with the primary antibody raised against GFP in guinea pig (0.5–2 µg/ml diluted in 0.1 M TBS with 1% NGS; Frontiers Institute, Japan), and incubated at 4 °C overnight. After washing, 1.4 nm gold particles conjugated to goat anti-guinea pig antibodies (diluted 1:100 in TBS buffer containing 2% NGS; Nanoprobes, NY) were added and incubated for 2 h. After several PB washes, sections were post-fixed in 1% glutaraldehyde prepared in the same buffer for 10 min. They were washed in double distilled water, followed by silver enhancement of the gold particles with an HQ Silver kit (Nanoprobes). Finally, the gold-silver-labeled sections were processed for electron microscopy as described previously^45^.

### Re-isolation of *Micromonospora* from legumes

Following the same procedure as above, five plants from each legume (*Lupinus*, *Medicago*, and *Trifolium*) were used to re-isolate the non-transformed *Micromonospora* strains Lupac 08 and Lupac 09^T^ after 3 weeks incubation. After counting the nodules from each plant, ten randomly chosen nodules (from the pool of 5 plants) were selected for bacterial isolation. Washed nodules were sterilized and processed as described previously and then the bacterial suspension was inoculated on yeast mannitol agar for isolation^10^. After counting the number of colonies having a *Micromonospora*-like appearance, several purified colonies were selected for DNA extraction and 16S rRNA gene sequencing for identification^10^. The sequences obtained were compared to the sequences deposited in the public databases (Lupac 08, AJ783992; Lupac 09^T^, AJ783993).
